# The value of C-reactive protein and lactate in the acute abdomen in the emergency department

**DOI:** 10.1186/1749-7922-7-22

**Published:** 2012-07-16

**Authors:** Zainna C Meyer, Jennifer MJ Schreinemakers, Lijckle van der Laan

**Affiliations:** 1 Department of Surgery, Amphia Hospital, Breda, the Netherlands

**Keywords:** Acute abdomen, C-reactive protein, Lactate

## Abstract

**Case presentation:**

This report describes the presentation of three critically ill patients with non-traumatic acute abdominal pain and increased concentrations of the biomarkers C-reactive protein (CRP) and lactate. In these three patients an exploratory laparotomy was carried out. Remarkably, the laparotomy showed no intra-abdominal abnormalities. We discuss the usefulness of these biomarkers in practice and their influence on establishing a diagnose and making a decision to perform an intervention.

**Conclusion:**

We conclude that biomarkers lactate and CRP in patients with acute abdominal pain should only be used in adjunct to the history and clinical findings, as they are not specific and can be misleading in establishing a diagnosis. In addition, relying on these biomarkers may contribute to more diagnostic examinations and/or unnecessary invasive interventions (for example laparotomy).

## Background

Diagnosing patients who present in the emergency department with acute abdominal pain can be challenging. In addition to history taking and physical examination, clinicians often use laboratory tests and radiological examinations to exclude diagnoses that can mimic acute abdominal pain for example pneumonia. Physicians in the emergency department often base their decisions for consultation of the surgeon for a laparotomy on clinical presentation combined with biochemical abnormalities. Examples of those biochemical parameters are high concentrations of C-reactive protein (CRP) or lactate concentrations [1,2]. The question remains if these parameters are reliable to diagnose an acute abdomen. The pitfall of relying on laboratory values could lead to over treatment or under treatment.

This report presents three patients with non-traumatic acute abdominal pain and abnormal C-reactive protein and/or lactate concentrations with a negative laparotomy. Furthermore, we discuss the usefulness of these markers in practice and their contribution to establish a diagnosis by means of interventions in the emergency department.

## Case presentation

### First case

Our first case was of a 65 years-old man who presented in the emergency department (ED) of our tertiary health care institute with acute abdominal pain which irradiated to the back in combination with hypotension. He was recently admitted into an orthopedic ward for two days with lumbar pain due to discopathy of all lumbar vertebrae with signs of spondylodicitis. Suspicion of an aneurysm of the abdominal aorta raised at presentation and a CT-scan was made. No acute pathology was seen except a dilatation of the stomach and small intestine. Laboratory results showed a leucocytes count of 8.4·10^9^/L (normal reference value: 4–10 ·10^9^/L), CRP concentration of 661 mg/L (0.8-2 mg/L), creatinine level of 548 μmol/L (45–100 μmol/L) with a glomerular filtration rate of 9 mL/min/1.73 m^2^ and a lactate level of 3.9 mmol/L (<1.8 mmol/L). Additional conventional chest X-rays was also made without pathological findings. Based on the clinical presentation and laboratory results we performed a laparotomy, which showed no abnormalities. He was admitted into the Intensive Care Unit (ICU) for pulmonary and cardiovascular support.

During the first five days of admission he was septic and required cardiovascular and pulmonary support. Continuous Venovenous Hemofiltration (CVVH) for acute kidney failure was started. The first blood cultures showed a staphylococcus aureus. At that time, the patient was treated with Tobramycine and Cefotaxim as prophylaxis for ventilator-associated pneumonia in combination with Orabase protective paste. A Positron Emission Tomography- Computed Tomography scan (PET-CT scan) and several CT-scans were performed, but did not show a focus.

**Figure 1:**
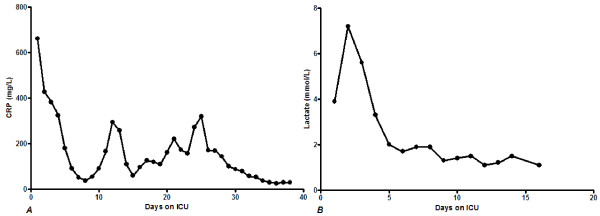
**C-reactive protein and lactate concentrations over time of the first case**. ***A*** C-reactive protein concentrations and ***B*** Lactate concentrations. C-reactive protein levels and lactate concentrations decreased to near normal values during the ICU stay.

After a stay on the ICU of one month with several complications he stabilized and was discharged. Complications included re-intubation, a central venous line infection with Enterococcus Faecium, an ischemic cerebrovascular accident in the left fronto-occipital region, an ileus and a segmental ischemic colitis with deep ulcers in the transverse colon. The lactate levels and CRP concentrations decreased to near normal values (Figure 1). Within a few days on the ward he developed a pneumosepsis, which was treated with Augmentin. When the patient deteriorated he was abstained from further treatment after consultation with patient and family. He deceased within 24 hours.

### Second case

The second patient was a 60 years-old woman. She presented in the ED with acute intense pain in the lower abdomen. One day earlier she started vomiting. Within the last six months she had several attacks of abdominal pain. The medical history included a laparoscopic cholecystectomy. On physical examination she had a tachycardia and was tachypnoeic. The lower abdomen was tender and a mass was palpated. A rectal and vaginal exam showed no abnormalities. Laboratory results demonstrated a leucocytes count of 18.1·10^9^/L, CRP 4 mmol/L and no abnormal kidney or liver function parameters. Arterial gas showed a pH of 7.71 (normal ref. values: 7.35-7.45), pCO_2_ of 1.7 kPa (4.7-6.4 kPa), pO_2_ 15.2 kPa (10.0-13.3 kPa), bicarbonate 4 mmol/L (22–29 mmol/L), base excess of −21.6 mmol/L (−3.0-3.0 mmol/L) and lactate level 6.7 mmol/L. Abdominal ultrasonography and conventional chest X-rays showed no abnormalities except a bladder retention which was treated.

Based on clinical and laboratory findings, a laparotomy was performed with the differential diagnosis of acute mesenterial ischemia. The laparotomy was negative for mesenterial ischemia, but bladder retention of more than one liter was found despite earlier treatment with an urinary catheter.

**Figure 2:**
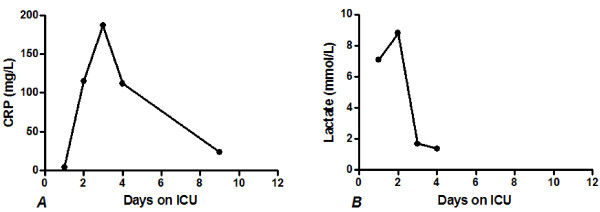
**C-reactive protein and lactate concentrations over time of the second case.** A C-reactive protein concentrations and B Lactate concentrations **A** C-reactive protein concentrations and **B** Lactate concentrations**.** After admittance into the ICU, the lactate levels increased till 10 mmol/L and thereafter decreased to normal values. The C-reactive protein levels follow the same pattern.

Postoperatively, the patient was admitted into the ICU and the lactate levels increased till 10 mmol/L and thereafter decreased to normal values (Figure 2). The CRP followed the same pattern (Figure 2). She was hemodynamically stable with low dosage of vasoactive medication and had mechanical ventilation support for a short period. Also, she developed acute kidney failure. Spontaneous mild correction of renal failure was seen within some days with a normal urine production of 60 ml/hour after administration of Furosemide. Abdominal pains in the right lower abdomen without a focus remained her main complain. After 3 days she was discharged from the ICU.

Complementary diagnostic examination by means of a gastroscopy showed a mild gastritis. A new abdominal ultrasonography showed no pathological findings.

During the stay on the internal medicine ward a spontaneous recovery of kidney failure was seen and constipation was successfully treated with Movicolon (a polyethylene glycol preparation; PEG 3350). Her abdominal pain decreased but was not totally over. After 11 days of admission, she was discharged.

### Third case

The third patient was a 68 years-old male which presented in the ED with a productive cough, sore throat and perspiration at night without a fever. Furthermore he developed a generalized rash. He recently spent time abroad (Finland) for construction work. Clinical features at the ED showed petechial rash on the face, extremities and abdomen. Furthermore, an enlarged submandibular lymph node was palpated. Examination of the abdomen was normal without tenderness. Laboratory results demonstrated a thrombocytes count of 20·10^9^/L (normal ref. values: 150-40010^9^/L), hemoglobin concentration of 9.1 mmol/L, leucocytes count of 6.6 mmol/L, CRP 9 mmol/L, bilirubine 24 μmol/L (0.0-20.0 μmol/L), alanine transaminase 212 U/L (0–45 U/L), aspartate transaminase 340 U/L (0–35 U/L), lactate dehydrogenase 677 U/L (,<248 U/L) alkaline phosphatase 684 U/L (0–115 U/L) and gamma-glutamyl transferase 265 U/L (0-55U/L). He was admitted into the internal medicine ward for further analysis of thrombocytopenia and liver failure.

Complementary diagnostic examination of the bone marrow demonstrated an increase in small lymfoide T-cells. Serology for viruses was negative. Conventional chest X-rays showed peribronchial changes like seen in COPD without other pathologic signs. Abdominal ultrasonography demonstrated a hepatomegaly, a small liver hemangioma and a thickened gallbladder wall without gallstones or signs of cholecystitis. Based on these findings the diagnosis for viral infection or auto-immune disease was made.

**Figure 3:**
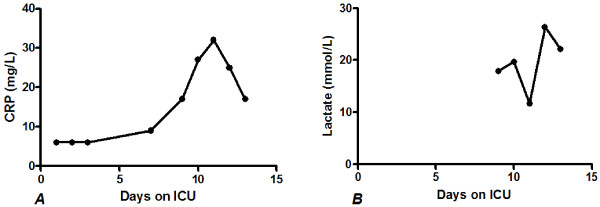
**C-reactive protein and lactate concentrations over time of the third case**. ***A*** C-reactive protein concentrations and ***B*** Lactate concentrations. During admission both C-reactive protein as lactate levels increased over time.

On the seventh day after admission he developed a fever of 38 °C without any complaints. The same generalized petechial was observed without abdominal tenderness. Laboratory results showed further liver failure and no signs of infection. Because of a fever (>39 °C), a CT-thorax and abdomen were made which showed a small consolidation in the right dorsal lung sinus, ascitis and infiltrative changes in mesenterium with air bubbles. It was suggested that these findings might indicate a bile-induced peritonitis. Antibiotics by means of Augmentin were started and a surgeon was consulted. Considering that the patient had no abdominal pain and no tenderness during physical examination, the team agreed to a conservative treatment. During the day and night the patient deteriorated with abnormal breathing, tachycardia of 110 beats per minute and jaundice without abdominal complaints or tenderness. New laboratory findings showed an increased lactate level with deterioration of liver tests (Figure 3). He was admitted into the ICU with the diagnosis abdominal sepsis with high lactate concentrations (lactate 15.1 mmol/L). The surgeon was consulted again based on a suspicion of intestinal pneumatosis due to acute mesenterial ischemia by means of high lactate levels, although no abdominal pain or abnormal physical examination was seen. A diagnostic laparotomy was performed. No pathological findings were observed except serosangulent fluids. He returned to the ICU.

On the ICU the patient remained hemodynamically unstable with high doses of inotropics and vasoactive medications. He had no abdominal pain and a normal physical examination. All cultures of blood, urine, sputum, ascitis and perioperative fluids were negative for infection. Nevertheless, broad spectrums of antibiotics were administered (Tobramycine, Augmentin and Doxycicline). CVVH was started due to acute kidney failure. During the next days the patient remained septic with high lactate concentrations, liver failure and kidney failure, disseminated intravascular coagulation accompanied with bleeding of the eyes and mucous membranes. Based on all the clinical findings, multi organ failure and inability to stabilize him, further treatment was stopped. He died 13 days after admission.

## Discussion

Although some biomarkers like lactate and C-reactive protein can be useful in the diagnosis of an acute abdomen, these cases demonstrate that these parameters can mislead the physician and contribute to more diagnostic examinations or unnecessary invasive interventions like a laparotomy.

As described in all cases the main suspicion was acute mesenteric ischemia. This is a complex disease with a high mortality rate [3]. Until now, no reliable parameters to help diagnose such serious disease have been found and a search to identify this factor continues. One of the markers that are frequently used is plasma lactate concentration. An increase of lactate levels indicates an anaerobe glucogenesis and therefore it is a parameter for inadequate perfusion, oxygenation and an estimate of tissue oxygen deficiency. Increased plasma lactate concentrations were observed in patients with mesenteric ischemia with a sensitivity of 100% and a specificity of 42% [3]. Yet, another study on patient with an acute abdomen and increased lactate levels in the ED, showed a sensitivity of 75% and specificity 39% when using lactate concentrations for the diagnosis of acute mesenteric ischemia [4]. On the other hand the study of Lange et. al. [3] showed that elevation in lactate concentration can be due to other conditions as well. For example general bacterial peritonitis and in about 50% of the cases with strangulated intestinal obstructions [3].

Furthermore, other conditions correlated with high lactate concentrations are (septic) shock, diabetic ketoacidosis, liver coma, renal failure and acute pancreatitis. When other conditions have been excluded, an increased lactate level often may indicate an emergency abdominal condition. Some authors recommend an laparotomy in all patients with abdominal complaints and a raised plasma lactate level when other conditions correlated with increased lactate levels have been excluded [3]. However, we believe that this matter is more subtle as we observe that lactate levels are being used as a parameter only for acute mesenterial ischemia. Our third case is an example of a patient without abdominal pain but with high lactate levels, probably due to liver failure. Based on the lactate levels, an unnecessary invasive diagnostic intervention, a laparotomy was performed. As a study concluded, the determination of lactate concentrations has no better sensitivity in establishing the diagnosis of patient with acute abdomen compared to clinical findings and normal laboratory examination [4].

Another biomarker often used in the emergency department to aid in the diagnosis of an acute abdomen is the C-reactive protein (CRP). Most studies have focused mainly on the use of this parameter in establishing the diagnosis of appendicitis. Few studies have assessed its diagnostic role in the general conditions describing acute abdominal pain. The diagnostic value of CRP in the overall patient with acute abdominal pain showed a sensitivity of 79%, specificity of 64% and global accuracy of 73% for predicting subsequent hospitalization using a cut-off value for positive test of >5 mg/L [2]. More recently, Salem et. al. [5] reviewed the diagnostic value of CRP in true surgical patients with acute abdominal pain in the ED. They concluded that CRP alone is not useful in differentiating between surgical causes of acute abdomen or self-limiting condition [5]. In addition, CRP can neither differentiate between surgical conditions requiring intervention from those who can be treated non-operatively [5]. In conclusion, these studies confirm the difficulty to diagnose an acute abdomen and assessing the need for a laparotomy as in our cases. Although high CRP levels or increase in CRP concentrations are seen in combination with abdominal complaints, it does not directly mean that a surgical complication should be the problem.

When CRP is compared with lactate, one study concluded that CRP is as a poor marker for the diagnosis of an acute abdomen considering that its activation is later in the onset of the disease compared to lactate or Interleukin-6 (IL-6) [1,5]. Patient with severe sepsis and those with sepsis on the ED with an acute abdomen can superiorly be differentiated by levels IL-6 and lactate [1]. But this study only included patients with sepsis or shock.

From our cases and a review of literature it is clear that we need more reliable markers to help establishing a fast and reliable diagnosis of patients with acute abdominal pain. Recently, the newer biomarker procalcitonin (PCT) showed to be a reliable marker to differentiate bacterial from nonbacterial infection or noninfectious inflammation with high accuracy [6]. Prospective studies on the use of PCT as screening test for appendicitis on the ED showed that this marker may only be useful in identifying patients with complicated (severe) appendicitis [7,8]. Furthermore, procalcitonin has also been proven to be helpful during the diagnosis or exclusion of acute mesenterial ischemia, intestinal ischemia or necrosis in acute bowel obstruction and abdominal sepsis [9-11]. Its use may be considered as additional tool to improve clinical decision making and appropriate therapy.

Imaging modalities have proven to be valuable adjuncts in diagnosis patients with acute abdominal pain. In one patient the CT-scan revealed no abnormalities and neither did the following laparotomy. The third patient did not have abdominal pain and the CT-scan showed potential bile peritonitis. The critical illness of the patient with abnormal increase in CRP and lactate concentration pushed the surgeons to perform a laparotomy, again without abnormalities. Perhaps, it should be recommended that all patients with acute abdominal pain and increased CRP and/or lactate levels should additionally undergo a CT-scan [12]. Even though it is known that a CT-scan is not completely reliable, a biphasic CT angiography has been reported with a sensitivity of approximately 90% for the diagnosis of mesenteric ischemia independent of the underlying pathology [13,14]. In addition, the CT scan can also provide alternative diagnoses for patients with an acute abdomen [13].

## Conclusion

These cases demonstrate that although biomarkers CRP and lactate can be useful in the diagnosis of an acute abdomen, they are not specific and can be misleading in establishing a diagnosis. In addition, relying on these biomarkers may contribute to more diagnostic examinations and/or unnecessary invasive interventions (e.g. laparotomy). We conclude that lactate levels and CRP concentrations in patients with acute abdominal pain should only be used in adjunction to the history and clinical findings and perhaps to a CT-scan as well.

## Consent

Written informed consent was obtained from all patients or next of kin of the patients for publication of this Case report. A copy of the written consent is available for review by the Editor-in-Chief of this Journal.

## Abbreviations

CRP, C-reactive Protein; ED, Emergency Department; ICU, Intensive Care Unit; CVVH, Continuous Venovenous Hemofiltration; CT-scan, Computed Tomography scan; PET-CT scan, Position Emission Tomography-Computed Tomography scan; PCT, Procalcitonin.

## Competing interests

The authors declare that they have no competing interests.

## Author’s contributions

ZM acquired data for the case report, interpreted the data, drafted the manuscript and has given approval for the final version. JM and LL interpreted the data, revised the manuscript critically for important intellectual content. All authors read and approved the version to be published.

## References

[B1] Ravishankaran P Shah AM Bhat R Correlation of interleukin-6, serum lactate, and C-reactive protein to inflammation, complication, and outcome during the surgical course of patients with acute abdomen J Interferon Cytokine Res 2011 31 685 690 10.1089/jir.2011.0021 21923250

[B2] Chi CH Shiesh SC Chen KW Wu MH Lin XZ C-reactive protein for the evaluation of acute abdominal pain Am J Emerg Med 1996 14 254 256 10.1016/S0735-6757(96)90169-2 8639195

[B3] Lange H Jackel R Usefulness of plasma lactate concentration in the diagnosis of acute abdominal disease Eur J Surg 1994 160 381 384 7948358

[B4] Vahl AC Out NJ Kapteijn BA Koomen AR Nothing gained from the determinations of plasma lactate levels in the evaluation of a patient with acute abdomen Ned Tijdschr Geneeskd 1998 142 901 904 9623186

[B5] Salem TA Molloy RG O'Dwyer PJ Prospective study on the role of C-reactive protein (CRP) in patients with an acute abdomen Ann R Coll Surg Engl 2007 89 233 237 17394705 10.1308/003588407X168389 PMC1964747

[B6] Becker KL Snider R Nylen ES Procalcitonin assay in systemic inflammation, infection, and sepsis: clinical utility and limitations Crit Care Med 2008 36 941 952 10.1097/CCM.0B013E318165BABB 18431284

[B7] Sand M Trullen XV Bechara FG Pala XF Sand D Landgrafe G Mann B A prospective bicenter study investigating the diagnostic value of procalcitonin in patients with acute appendicitis Eur Surg Res 2009 43 291 297 10.1159/000232939 19672084 PMC2790741

[B8] Wu JY Chen HC Lee SH Chan RC Lee CC 2012 Diagnostic Role of Procalcitonin in Patients with Suspected Appendicitis. World J Surg, Chang SS [Epub ahead of print] 10.1007/s00268-012-1579-z 22491817

[B9] Markogiannakis H Memos N Messaris E Dardamanis D Larentzakis A Papanikolaou D Zografos GC Manouras A Predictive value of procalcitonin for bowel ischemia and necrosis in bowel obstruction Surgery 2011 149 394 403 10.1016/j.surg.2010.08.007 20869092

[B10] Rau B Kruger CM Schilling MK Procalcitonin: improved biochemical severity stratification and postoperative monitoring in severe abdominal inflammation and sepsis Langenbecks Arch Surg 2004 389 134 144 10.1007/s00423-004-0463-1 15007651

[B11] Ivancevic N Radenkovic D Bumbasirevic V Karamarkovic A Jeremic V Kalezic N Vodnik T Beleslin B Milic N Gregoric P Zarkovic M Procalcitonin in preoperative diagnosis of abdominal sepsis Langenbecks Arch Surg 2008 393 397 403 10.1007/s00423-007-0239-5 17968584

[B12] Mahler CW Boermeester MA Stoker J Obertop H Gouma DJ Diagnostic modalities in diagnosis of adult patients with acute abdominal pain Ned Tijdschr Geneeskd 2004 148 2474 2480 15638193

[B13] Furukawa A Kanasaki S Kono N Wakamiya M Tanaka T Takahashi M Murata K CT diagnosis of acute mesenteric ischemia from various causes AJR Am J Roentgenol 2009 192 408 416 10.2214/AJR.08.1138 19155403

[B14] Kirkpatrick ID Kroeker MA Greenberg HM Biphasic CT with mesenteric CT angiography in the evaluation of acute mesenteric ischemia: initial experience Radiology 2003 229 91 98 10.1148/radiol.2291020991 12944600

